# Energy loss evaluation of axial flow pump systems in reverse power generation operations based on entropy production theory

**DOI:** 10.1038/s41598-022-12667-7

**Published:** 2022-05-23

**Authors:** Xiaowen Zhang, Fangping Tang

**Affiliations:** College of Hydraulic Science and Engineering, Yangzhou University, Yangzhou, 225009 China

**Keywords:** Energy infrastructure, Mechanical engineering

## Abstract

The use of existing large pumping station equipment for upstream residual water reverse power generation is an unrealized yet valuable renewable energy project. At present, some large axial flow pump stations have begun to perform reverse power generation operations; however, related research has not yet started. In this paper, entropy generation theory is applied to a large-scale axial flow pump station system in reverse power generation operations, and the entropy generation method is used to investigate the accurate size and distribution of the mechanical energy dissipation of each component under different flow conditions. First, the energy characteristics and pressure fluctuations in the pump of the large axial flow pump station system are experimentally tested under reverse power generation conditions. The reliability of the entropy generation numerical calculation is verified both experimentally and theoretically. Then, the proportion of each component in the total entropy production is compared to illustrate how each component contributes to the total entropy production of the system and how this contribution changes as operating conditions vary. Then, the type of entropy production of each component is accurately determined under different flow conditions, revealing the changes in the proportions of the different types of entropy production of each component. Finally, components with large mechanical energy dissipations are selected, and the changes and causes of the energy dissipation distribution of the components are thoroughly analysed under different flow conditions. The research results can aid in better understanding the energy dissipation mechanism of large axial flow pump systems in reverse power generation operations.

## Introduction

In recent years, pump reversal has become an increasingly popular method for replacing turbine operations in engineering^1–6^, and many micro- and small hydropower plants in remote areas have started to use axial flow pump reversal to replace turbine operations^7–11^. Some large-scale axial flow pump station system administrators in China have found that reverse power generation using upstream inflow water can generate a large amount of clean energy while providing considerable economic benefits. Therefore, some attempts at reverse power generation using axial flow pump stations have been used in actual operations.

The current operation of various large axial flow pump stations for reverse power generation^12^ has shown that it is technically feasible to use the reverse operation of a large axial flow pump system to generate power. However, for the widespread use of axial flow pump stations for reverse power generation, the utilization rate of low-head water energy and the energy benefits offered by reverse power generation of large axial flow pump systems must first be investigated. This requires an in-depth study of the head loss and energy dissipation mechanisms of large axial flow pump systems in reverse power generation operations.

Researchers in China and abroad have studied the reverse power generation characteristics of axial flow pumps, mainly focusing on the optimization of the impeller of small axial flow pumps to improve the hydraulic performance of axial flow pumps operating as turbines in the Pico Hydropower Station^13,14^. To date, research on the energy characteristics and energy dissipation mechanisms of large axial flow pump systems in reverse power generation operations is still lacking.

The pressure drop method has been the most widely used method for studying hydraulic loss and energy dissipation in hydraulic machinery in recent decades. However, the pressure drop method cannot determine the specific location of energy dissipation or quantify energy dissipation. In recent years, some scholars have proposed introducing entropy production theory into rotating fluid machinery to evaluate energy dissipation mechanisms in fluid machinery^15–21^. Gong et al.^19^ first used entropy production theory to evaluate the internal flow loss of Francis turbines and determined the specific location and loss intensity of the internal flow loss in the turbines. Chang et al.^20^ applied entropy production theory to study the internal flow loss and energy dissipation mechanisms of a self-priming pump and optimized the blade profile of the self-priming pump based on the entropy production analysis results. Pei et al.^22^ designed six groups with different distances between the impeller and guide vane in a low head axial flow pump. Entropy production theory was used to study the energy dissipation in the axial flow pump under different schemes. It was found that turbulent dissipation dominated the mechanical energy consumption of the axial flow pump. Mohammad et al.^23^ analysed the energy dissipation mechanism of a small centrifugal pump powered by a reverse turbine under different flow conditions by using entropy production theory. The vortex at the inlet of the impeller and flow separation at the outlet of the impeller were found to be the main causes of entropy generation in the small centrifugal pump operated by the reverse turbine. The entropy production loss of the draft tube was most apparent in the entropy production loss of the PAT components.

In this work, entropy generation theory is used to reveal the energy dissipation mechanism of a large axial flow pump system in reverse power generation operations. The exact size and distribution of the mechanical energy dissipation of each component (inlet channel, bulb body, guide vane, impeller and outlet channel) under different flow conditions are studied with the entropy generation method. First, a high-precision full-feature hydraulic machinery test bench was built to investigate the energy characteristics and pressure fluctuations in the pump of a large axial flow pump station system under reverse power generation conditions. The reliability of the entropy production numerical calculation was verified by the experimental and theoretical results. Then, the proportion of entropy production of each component in the total entropy production is compared to illustrate how each component contributes to the total entropy production of the system and how this contribution changes as the operating conditions vary. Then, the entropy production types of each component are accurately determined under different flow conditions, and the change in the proportion of the different types of entropy production of each component in the total entropy production is revealed. Finally, components with large mechanical energy dissipations are selected, and the changes and causes of the energy dissipation distribution of the components are thoroughly analysed under different flow conditions.

## Theory of entropy production

According to the second law of thermodynamics, the loss of mechanical energy is irreversibly converted to internal energy, and this thermodynamic process eventually leads to an increase in entropy production. Within a hydraulic mechanical system, energy dissipation inevitably occurs during turbulent motion. Therefore, it is appropriate to use entropy production theory to study the internal hydraulic loss and energy dissipation mechanisms of hydraulic machinery^24^.

During turbulent motion in a hydraulic mechanical system, the water flow velocity includes the average velocity and the fluctuating velocity. The entropy generation during turbulent motion can also be divided into two components: the entropy generated by the time-averaged flow motion and the entropy generated by the dissipation of turbulent kinetic energy due to the velocity. Therefore, the local entropy production rate during turbulent motion can be calculated as follows:

1 $$ \mathop {S_{{\overline{D} }}^{\prime \prime \prime }}\limits^{ \cdot } = \mathop {S_{{\overline{D} }}^{\prime \prime \prime }}\limits^{ \cdot } + \mathop {S_{{D^{\prime } }}^{\prime \prime \prime }}\limits^{ \cdot } $$

The entropy production rate caused by the time-averaged motion of the water flow can be calculated as follows:

2 $$ \mathop {S_{{\overline{D} }}^{\prime \prime \prime }}\limits^{ \cdot } = \frac{{2\mu_{eff} }}{T}\left[ {\left( {\frac{{\partial \overline{{u_{1} }} }}{{\partial x_{1} }}} \right)^{2} + \left( {\frac{{\partial \overline{{u_{2} }} }}{{\partial x_{2} }}} \right)^{2} + \left( {\frac{{\partial \overline{{u_{3} }} }}{{\partial x_{3} }}} \right)^{2} } \right] + \frac{{\mu_{eff} }}{T}\left[ {\left( {\frac{{\partial \overline{{u_{2} }} }}{{\partial x_{1} }} + \frac{{\partial \overline{{u_{1} }} }}{{\partial x_{2} }}} \right)^{2} + \left( {\frac{{\partial \overline{{u_{2} }} }}{{\partial x_{3} }} + \frac{{\partial \overline{{u_{3} }} }}{{\partial x_{2} }}} \right)^{2} + \left( {\frac{{\partial \overline{{u_{3} }} }}{{\partial x_{1} }} + \frac{{\partial \overline{{u_{1} }} }}{{\partial x_{3} }}} \right)^{2} } \right] $$

The entropy production rate caused by turbulent kinetic energy dissipation due to the turbulent fluctuation velocity can be calculated as follows:

3 $$ \mathop {S_{{D^{^{\prime}} }}^{\prime \prime \prime }}\limits^{ \cdot } = \frac{{2\mu_{eff} }}{T}\left[ {\left( {\frac{{\partial u_{1}^{^{\prime}} }}{{\partial x_{1} }}} \right)^{2} + \left( {\frac{{\partial u_{2}^{^{\prime}} }}{{\partial x_{2} }}} \right)^{2} + \left( {\frac{{\partial u_{3}^{^{\prime}} }}{{\partial x_{3} }}} \right)^{2} } \right] + \frac{{\mu_{eff} }}{T}\left[ {\left( {\frac{{\partial u_{2}^{^{\prime}} }}{{\partial x_{1} }} + \frac{{\partial u_{1}^{^{\prime}} }}{{\partial x_{2} }}} \right)^{2} + \left( {\frac{{\partial u_{2}^{^{\prime}} }}{{\partial x_{3} }} + \frac{{\partial u_{3}^{^{\prime}} }}{{\partial x_{2} }}} \right)^{2} + \left( {\frac{{\partial u_{3}^{^{\prime}} }}{{\partial x_{1} }} + \frac{{\partial u_{1}^{^{\prime}} }}{{\partial x_{3} }}} \right)^{2} } \right] $$

4 $$ \mu_{eff} = \mu + \mu_{t} $$

where $\mathop {S_{{\overline{D} }}^{\prime \prime \prime }}\limits^{ \cdot }$ is the average entropy production rate, which is known as the direct dissipation term; $\mathop {S_{{D^{^{\prime}} }}^{\prime \prime \prime }}\limits^{ \cdot }$ is the entropy production rate of the velocity fluctuation, which is known as the turbulent dissipation term; $\mu_{eff}$ is the effective dynamic viscosity; $\mu$ is the turbulent viscosity; and $\mu_{t}$ is the turbulent dynamic viscosity.

$\mathop {S_{{\overline{D} }}^{\prime \prime \prime }}\limits^{ \cdot }$ can be obtained directly through numerical calculations, but the entropy production rate $\mathop {S_{{D^{^{\prime}} }}^{\prime \prime \prime }}\limits^{ \cdot }$ caused by the turbulent fluctuating velocity cannot be obtained directly through numerical calculations. However, according to previous work on local entropy generation theory, the entropy generation caused by the turbulent fluctuating velocity is closely related to the turbulence model *ε* or *ω* used in the numerical calculation. Therefore, in the SST *k*–*ω* turbulence model, the entropy production caused by the turbulent fluctuating velocity can be expressed as follows:

5 $$ \mathop {S_{{D^{^{\prime}} }}^{\prime \prime \prime }}\limits^{ \cdot } = \alpha \frac{\rho \omega k}{T} $$

where *α* is a constant with a value of 0.09. *ω* is the frequency of the turbulent vortex, s^−1^. *k* is the turbulence intensity, m^2^/s^2^.

In the calculation of the entropy production rate, because the entropy production rate has a strong wall effect, the entropy production rate near the wall cannot be ignored. The calculation formula for the entropy production rate near the wall is as follows:

6 $$ \mathop {S_{w}^{\prime \prime \prime }}\limits^{ \cdot } = \frac{{\overrightarrow {\tau } \cdot \overrightarrow {v} }}{T} $$

where $\overrightarrow {\tau }$ is the wall shear stress, Pa. $\overrightarrow {v}$ is the velocity near the wall, m/s.

Therefore, the sum of the entropy production in the whole hydraulic mechanical system is the integration of the local entropy production rate and the wall entropy production rate. The calculation formulas are as follows:

7 $$ \begin{aligned} & S_{{pro,\overline{D} }} = \int\limits_{v} {\mathop {S_{{\overline{D} }}^{\prime \prime \prime }}\limits^{ \cdot } } dV \\ & S_{{pro,D^{^{\prime}} }} = \int\limits_{v} {\mathop {S_{{D^{^{\prime}} }}^{\prime \prime \prime }}\limits^{ \cdot } } dV \\ & S_{pro,W} = \int\limits_{A} {\frac{{\overrightarrow {\tau } \cdot \overrightarrow {v} }}{T}} dA \\ & S_{pro} = S_{{pro,\overline{D} }} + S_{{pro,D^{^{\prime}} }} + S_{pro,W} \\ \end{aligned} $$

where $S_{{pro,\overline{D} }}$ is the entropy production caused by the mean velocity, namely, the direct dissipative entropy production; $S_{{pro,D^{^{\prime}} }}$ is the entropy production caused by the fluctuating velocity, namely, the turbulent dissipation entropy production; $S_{pro,W}$ is the entropy production at the wall, namely, the dissipation entropy production at the wall; *V* is the volume of the fluid domain; and *A* is the wall area of the fluid domain.

## Numerical methods and experimental systems

### Research object

**Figure 1:**
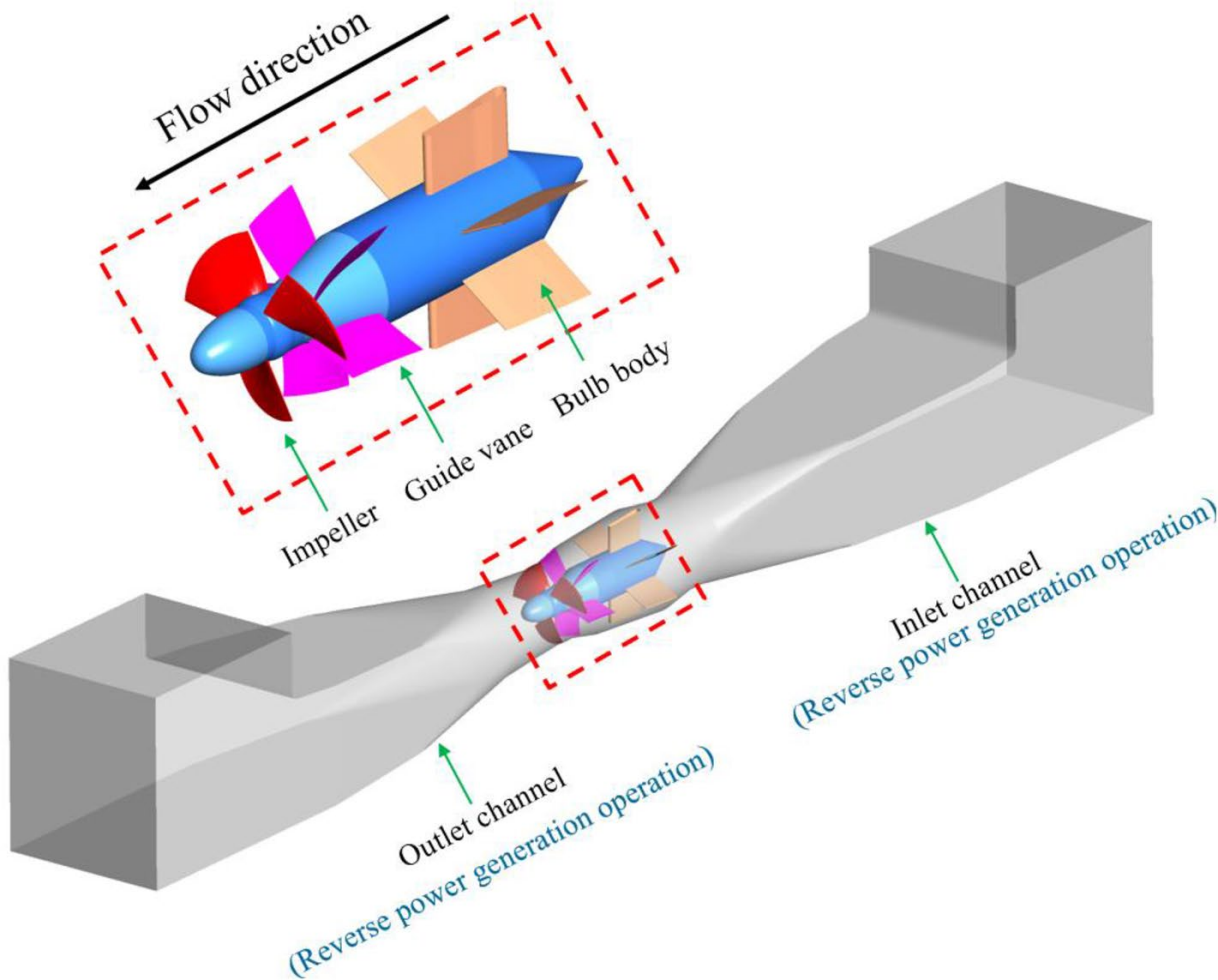
Three-dimensional model of a large axial flow pump system for reverse power generation operations.

In this paper, a horizontal axial flow pump station model in China is selected as the research object. The design head of the axial flow pump station model is 203 l/s; the design head is 2.01 m, and the efficiency of the design operating point is 68.39%. Table 1 shows the main geometric parameters of the axial flow pump hydraulic model equipped in the pump station system. Three-dimensional modelling of the large-scale axial flow pump system is carried out by UG software^25^. The calculation domain includes the inlet and outlet extension sections, inlet channel, outlet channel, impeller, guide vane, and bulb body. When the large axial flow pump system uses upstream inflow water for reverse power generation, the flow direction and impeller rotation direction in the system are opposite to those of conventional pumping conditions. Figure 1 shows a three-dimensional model of the large axial flow pump system for reverse power generation operations.

**Table 1:** Main geometric parameters of the axial flow pump hydraulic model.

Parameter	Symbol	Numerical value
Specific speed	n_s_	1179
Impeller diameter	D	300 mm
Tip clearance	C	0.15 mm
Blade placement angle	*β*_1_	0
Impeller blades number	Z_1_	3
Guide vane number	Z_2_	5

### Calculation grids

**Figure 2:**
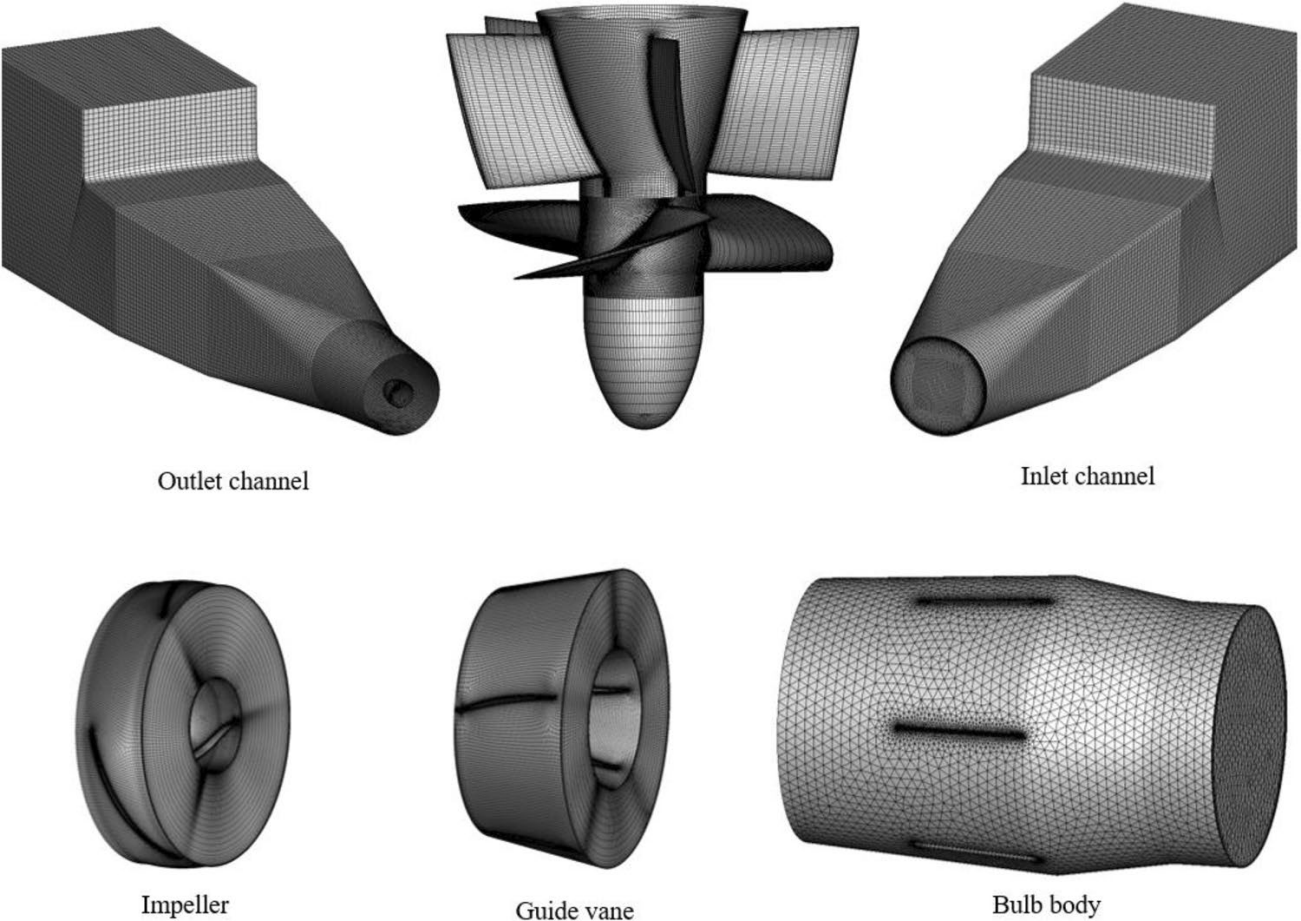
Calculation grids.

The calculation model is meshed based on ICEM software. The computational domain uses a hybrid grid for grid division^26^, in which the inlet channel, outlet channel, impeller, and guide vanes are structured grids, and the bulb body is an unstructured grid. During the meshing process, to control the influence of the grid topology and grid number on the numerical calculation results, the grid aspect ratio is controlled to ensure that the sizes of adjacent grid nodes are similar. In CFD calculations, the aspect ratio of the grid should be less than 10 ~ 100, and the grid in this paper satisfies this requirement. After the meshing process is completed, the edge walls of the impeller and other key positions are encrypted to ensure that the y + values of the impeller grid are within 10. When the optimal reverse power generation condition is taken as the representative condition in the grid independence test, the experimental head of the optimal reverse power generation condition is 3.85 m, and the experimental efficiency is 71.69%. The results of the grid independence test are shown in Table 2. Table 2 shows that when the grid number is greater than 5.09 × 10^6^, the head fluctuations of the large axial flow pump system model for reverse power generation tend to be stable. Considering the calculation accuracy and calculation cost, a grid number of 6.26 × 10^6^ was selected for the numerical calculation. The calculation grid is shown in Fig. 2.

**Table 2:** Grid independence test.

Scheme	Grid number of the inlet channel	Grid number of the impeller	Grid number of the guide vane	Grid number of the bulb body	Grid number of the outlet channel	Total grid number	*H* (*m*)
S1	0.34 × 10^6^	0.90 × 10^6^	0.49 × 10^6^	0.44 × 10^6^	0.26 × 10^6^	2.43 × 10^6^	3.32
S2	0.54 × 10^6^	1.59 × 10^6^	0.72 × 10^6^	0.66 × 10^6^	0.48 × 10^6^	3.89 × 10^6^	3.57
S3	0.76 × 10^6^	1.85 × 10^6^	0.96 × 10^6^	0.91 × 10^6^	0.61 × 10^6^	5.09 × 10^6^	3.65
S4	0.92 × 10^6^	2.21 × 10^6^	1.26 × 10^6^	1.15 × 10^6^	0.73 × 10^6^	6.26 × 10^6^	3.68
S5	1.09 × 10^6^	2.53 × 10^6^	1.37 × 10^6^	1.38 × 10^6^	0.87 × 10^6^	7.24 × 10^6^	3.69

### Turbulence model

The SST *k–ω* turbulence model is a two-equation eddy viscosity model that considers the turbulent shear force in the turbulent viscosity and can better predict unsteady flow characteristics such as vortices. Furthermore, the wall function of the SST *k–ω* turbulence model can accurately capture the viscosity characteristics of a low Reynolds number near the wall region of the impeller machinery, and the calculation results are more accurate in flows with pressure gradients. Therefore, the SST *k–ω* turbulence model was selected for the numerical calculations in this paper. The equations of the SST *k–ω* turbulence model are as follows:

8 $$ \frac{{\partial (\rho_{{\text{m}}} k)}}{\partial t} + \frac{{\partial \left( {\rho_{{\text{m}}} u_{j} k} \right)}}{{\partial x_{j} }} = \frac{\partial }{{\partial x_{j} }}\left[ {\left( {\mu_{{\text{m}}} + \frac{{\mu_{t} }}{{\sigma_{k} }}} \right)\frac{\partial k}{{\partial x_{j} }}} \right] + P_{k} - \beta^{*} \rho_{{\text{m}}} \omega k $$

9 $$ \frac{{\partial (\rho_{{\text{m}}} \omega )}}{\partial t} + \frac{{\partial \left( {\rho_{{\text{m}}} u_{j} \omega } \right)}}{{\partial x_{j} }} = \frac{\partial }{{\partial x_{j} }}\left[ {\left( {\mu_{{\text{m}}} + \frac{{\mu_{t} }}{{\sigma_{\omega } }}} \right)\frac{\partial \omega }{{\partial x_{j} }}} \right] + \frac{\alpha \omega }{k}P_{k} - D_{\omega } + Cd_{\omega } $$

10 $$ \left. \begin{gathered} \mu_{t} = \frac{{\rho_{{\text{m}}} \alpha_{1} k}}{{\max \left( {\alpha_{1} \omega ,SF_{2} } \right)}} \hfill \\ D_{\omega } = \beta \rho_{{\text{m}}} \omega^{2} ,C = 2\rho_{{\text{m}}} \left( {1 - F_{1} } \right) \hfill \\ d_{\omega } = \frac{1}{{\omega \sigma_{\omega 2} }}\frac{\partial k}{{\partial x_{j} }}\frac{\partial \omega }{{\partial x_{j} }} \hfill \\ \end{gathered} \right\} $$

where $k$ is the turbulent kinetic energy. ${\upomega }$ is the turbulence frequency. $P_{k}$ is the turbulence production rate. $\rho_{m}$ is the mixture density, kg/m^3^. $u_{j}$ is the velocity component in the j direction. $\mu_{t}$ is the turbulence viscosity, and $\mu$ is the dynamic viscosity, Pa s. $F_{1}$ and $F_{2}$ are mixed functions. $\beta^{*}$, ${\upbeta }$, ${\upalpha }$, $\alpha_{1}$, $\alpha_{k}$, $\sigma_{\omega }$, and $\sigma_{\omega 2}$ are all empirical coefficients. $S$ is the invariant of the strain rate. $D_{\omega }$ is the dissipation term in the ${\upomega }$-equation. $Cd_{\omega }$ is the cross-diffusion term in the SST model.

### Numerical settings

The numerical calculation is performed in the fluid calculation software ANSYS CFX17.0. In the unsteady calculation process, the steady calculation results are used as the initial file, the rotor and stator are coupled by the frozen rotor method, and the transient frozen rotor slip interface is used for information transmission between the rotating and static domains. The inlet boundary condition of the calculation domain is set as a mass flow inlet, and the outlet boundary condition is set as an opening outlet with a relative pressure of 0 Pa. All walls in the calculation domain are set to no slip conditions. The maximum number of iterations for each time step of the unsteady calculation is set to 20, and the residual convergence limit is set to 1 × 10^−5^. The time step of the unsteady calculation is set to 5 × 10^–4^ s, and each impeller rotation cycle is sampled 120 times. Thus, the total calculation time is 0.96 s for 16 impeller rotation cycles. The first eight impeller rotation periods ensure the stability of the calculation. The remaining eight impeller rotation periods are selected for variable analysis. The variables used in this paper are the average variables of the last eight cycles.

### Experimental test system

**Figure 3:**
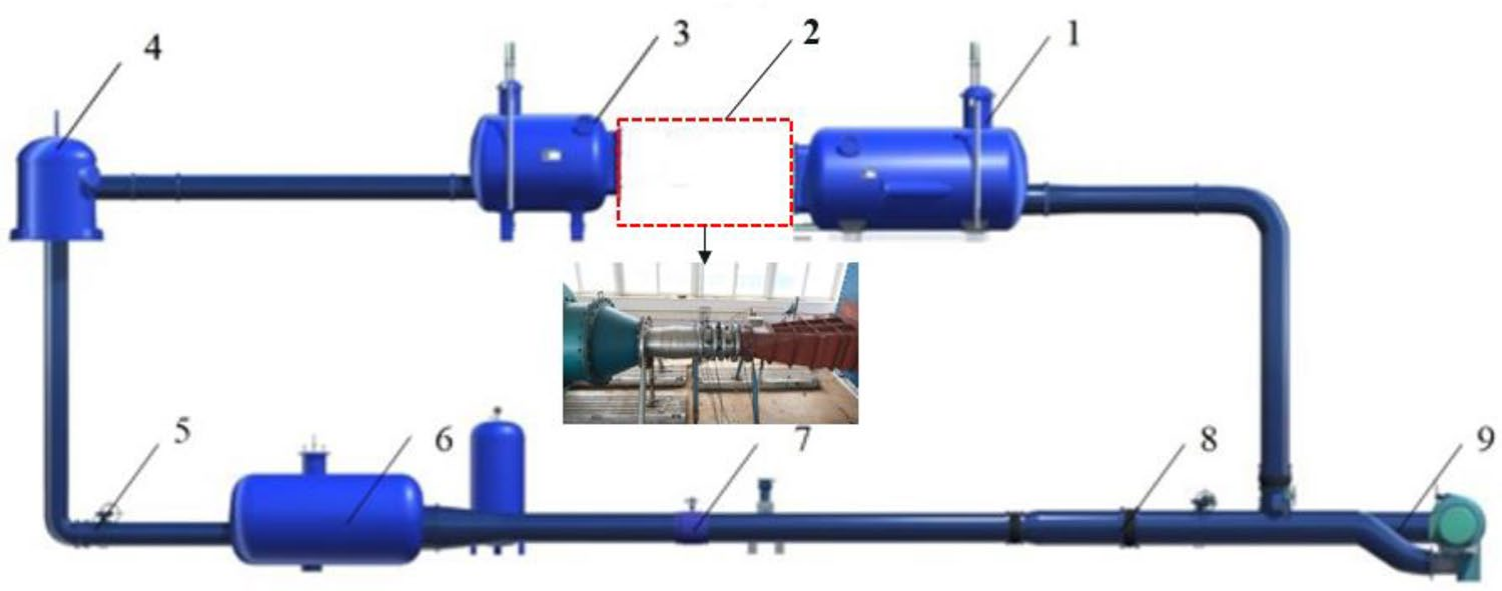
Physical schematics of the test bench. 1. Intake tank, 2. Tested pump unit and drive motor, 3. Pressure outlet tank, 4. Bifurcation tank, 5. Condition-regulating gate valve, 6. Voltage-regulating rectifier, 7. Electromagnetic flowmeter, 8. System forward and reverse operation control gate valve, 9. Auxiliary pump unit.

**Figure 4:**
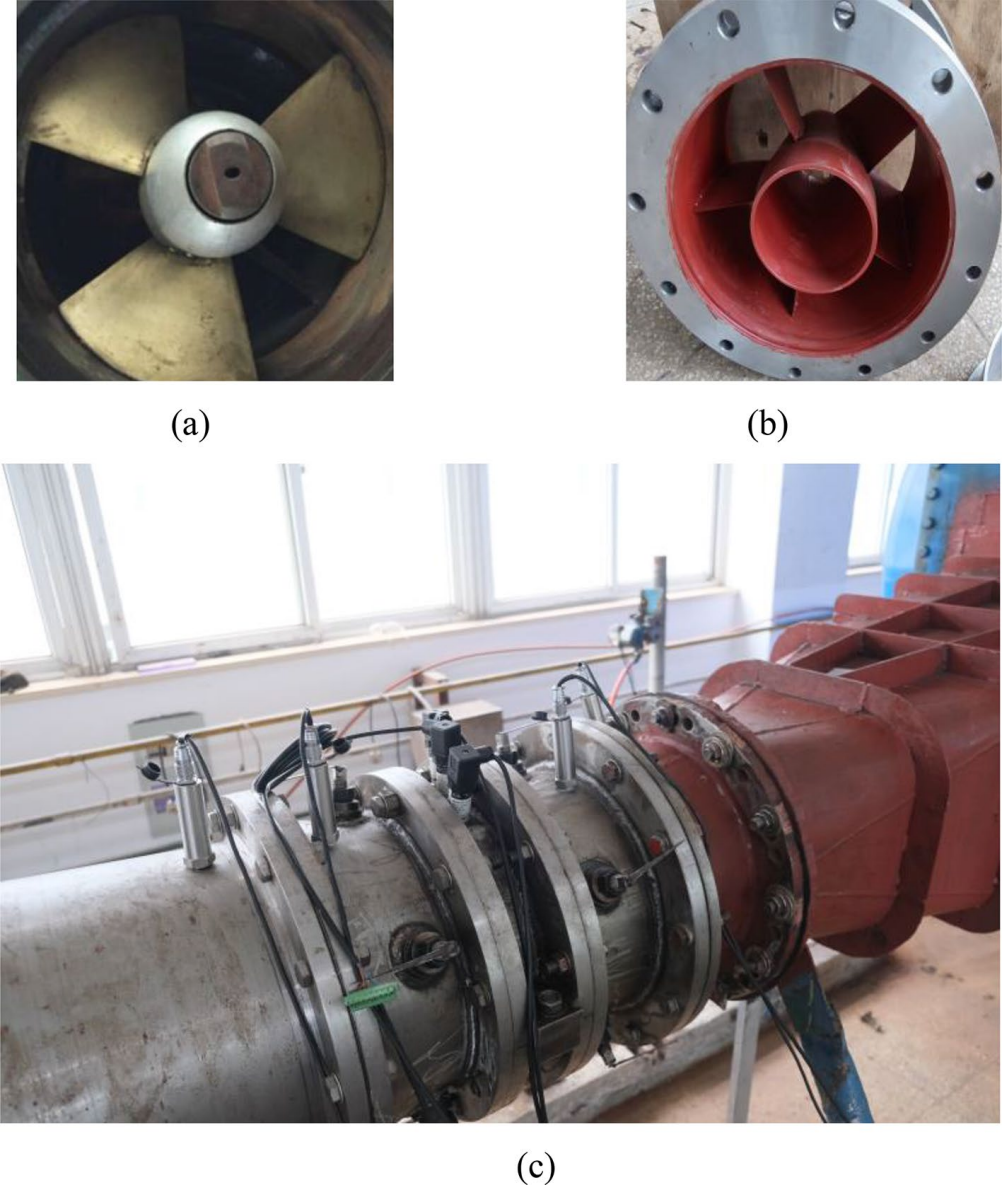
Experimental model of the large axial flow pump station system. (**a**) Impeller, (**b**) Guide Vane, (**c**) Installation diagram of the pump station model.

In this paper, a high-precision hydraulic machinery full-feature test bench is built. The energy characteristics and pressure fluctuations in the pump of the large axial flow pump station system model can be measured under reverse power generation conditions on the test bench. The schematic diagram of the experimental system is shown in Fig. 3. The specific parameters of the experimental system are shown in Table 3. The experimental model of large axial flow pump station system is shown in Fig. 4. This experiment has good repeatability, and the maximum head error in the energy characteristic test is less than 0.5%. In the actual test process, 16 different flow conditions for the axial flow pump system for reverse power generation operations were tested according to the requirements of the ‘acceptance test specification for the pump model and device model (SL 140–2006)’. During the experiment, the pressure pulsation acquisition and energy characteristic tests were carried out simultaneously. The pressure pulsation monitoring point was located on the outer wall of the water pump, and the axial position was located in the middle of the impeller.

**Table 3:** Specific parameters of the main measuring instruments.

Measurement items	Instrument name	Instrument types	Instrument range	Calibration accuracy (%)
Head	Difference pressure transmitter	EJA 110A	0 ~ 200 kPa	± 0.1
Flow	Electromagnetic flowmeter	E-mag type	DN400 mm	± 0.20
Torque	Torsiograph	JW-3	200 N·m	± 0.15
Rotation speed	Speed and torque sensor	JC	0 ~ 10,000 r/min	± 0.15

## Results and discussion

### Verification of the numerical calculation

**Figure 5:**
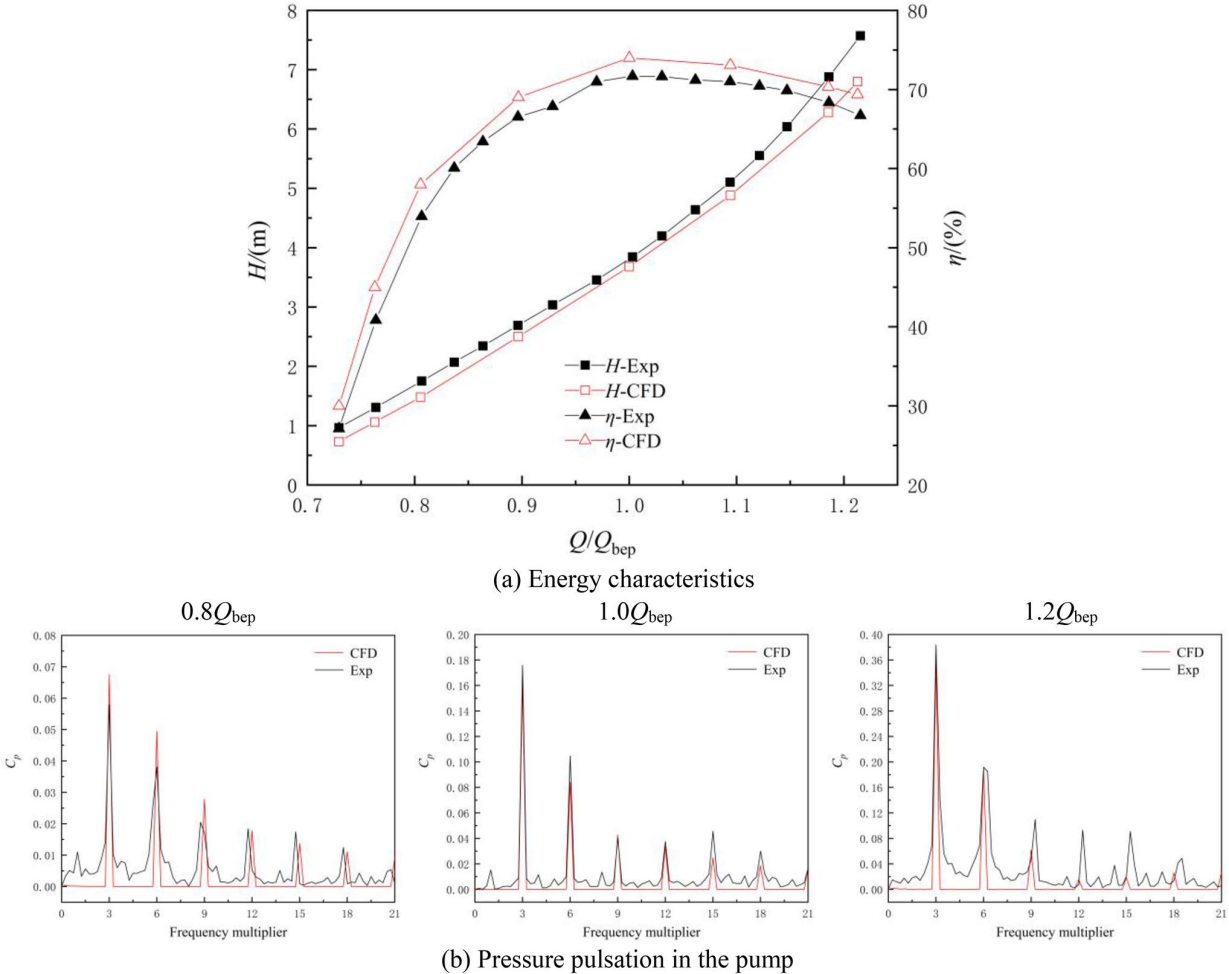
Comparison between numerical results and experimental results.

To verify the reliability of the numerical calculation, the large axial flow pump system model was tested under reverse power generation conditions at an experimental speed of 1000 r/min. The energy characteristics and internal pressure pulsation characteristics of the axial flow pump system under reverse power generation conditions are shown in Fig. 5. The pressure pulsation coefficient *C*_*p*_ in Fig. 5b is defined as follows:

11 $$ C_{p} = \frac{{p - \overline{p} }}{{0.5\rho u_{2}^{2} }} $$

where *p* is the transient pressure value, $\overline{p}$ is the average pressure value, and *u*_2_ is the circumferential velocity of the impeller outlet.

Figure 5a shows that as the flow rate increases, the head of the pump system for reverse power generation gradually increases. The efficiency first increases and then decreases; however, it still maintains high hydraulic conversion efficiency under large flow conditions. The optimal head obtained by the numerical calculation is 3.68 m, and the corresponding efficiency is 74.01%. A comparison of the numerical and experimental results shows that the change trend of the numerically calculated energy characteristic curve is essentially consistent with the experimental energy characteristic curve, with a maximum error of approximately 5%. Figure 5b shows that the waveform of the pressure pulsation in the pump obtained by the numerical calculation is essentially consistent with that obtained in the experiment. The frequency component of the pressure pulsation is highly consistent with the experimental results; however, there is some error in the amplitude of the pressure pulsation. In general, the numerical calculation results are in good agreement with the experimental results; thus, high-precision predictions for the energy characteristics of the system and the transient flow in the system are possibly, verifying the accuracy of the subsequent calculations and analyses.

### Total entropy production distribution of the system

**Figure 6:**
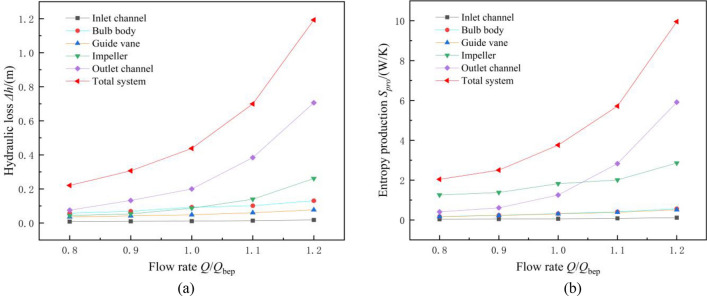
Distribution of the energy loss in the system under different flow conditions. (**a**) Distribution of the hydraulic loss in each region (pressure drop method), (**b**) Distribution of the total entropy production in each region (entropy production method).

Figure 6 shows the distribution of energy loss in the system under different flow conditions. Figure 6a shows the hydraulic loss distribution of each region obtained by the pressure drop method, while Fig. 6b shows the total entropy production distribution of each region obtained by the entropy production method. In Fig. 6a, $\Delta h$ can be calculated as follows:

12 $$ \Delta h = \frac{P2 - P1}{{\rho g}} $$

where $\Delta h$ is hydraulic loss, m. *P*2 and *P*1 are the total export pressure and total import pressure of the flow components, Pa. $\rho$ is the density of water, kg/m^3^.

Figure 6 shows that as the flow rate increases, the hydraulic loss and total entropy production in each region gradually increase; however, the sensitivity of the energy loss in each region to the increase in flow rate differs. The energy losses of the inlet, bulb and guide vane are less sensitive to the increase in flow rate, while the outlet is more sensitive to the increase in flow rate. The energy loss of the outlet increases significantly with increasing flow rate. For a flow rate of 0.8*Q*_bep_, the hydraulic loss of the outlet is 0.076 m, and the total entropy production value is 0.404 W/K. For a flow rate of 1.0*Q*_bep_, the hydraulic loss of the outlet is 0.199 m, and the total entropy production value is 1.252 W/K. For a flow rate of 1.2*Q*_bep_, the hydraulic loss of the outlet is 0.706 m, and the total entropy production value is 5.911 W/K. Figure 6a and b show that the total entropy production distribution in each region is essentially consistent with the variation trend of the hydraulic loss distribution in each region, showing that the entropy production method used in this paper can also be used to evaluate the energy loss of large axial flow pump systems under reverse power generation conditions.

**Figure 7:**
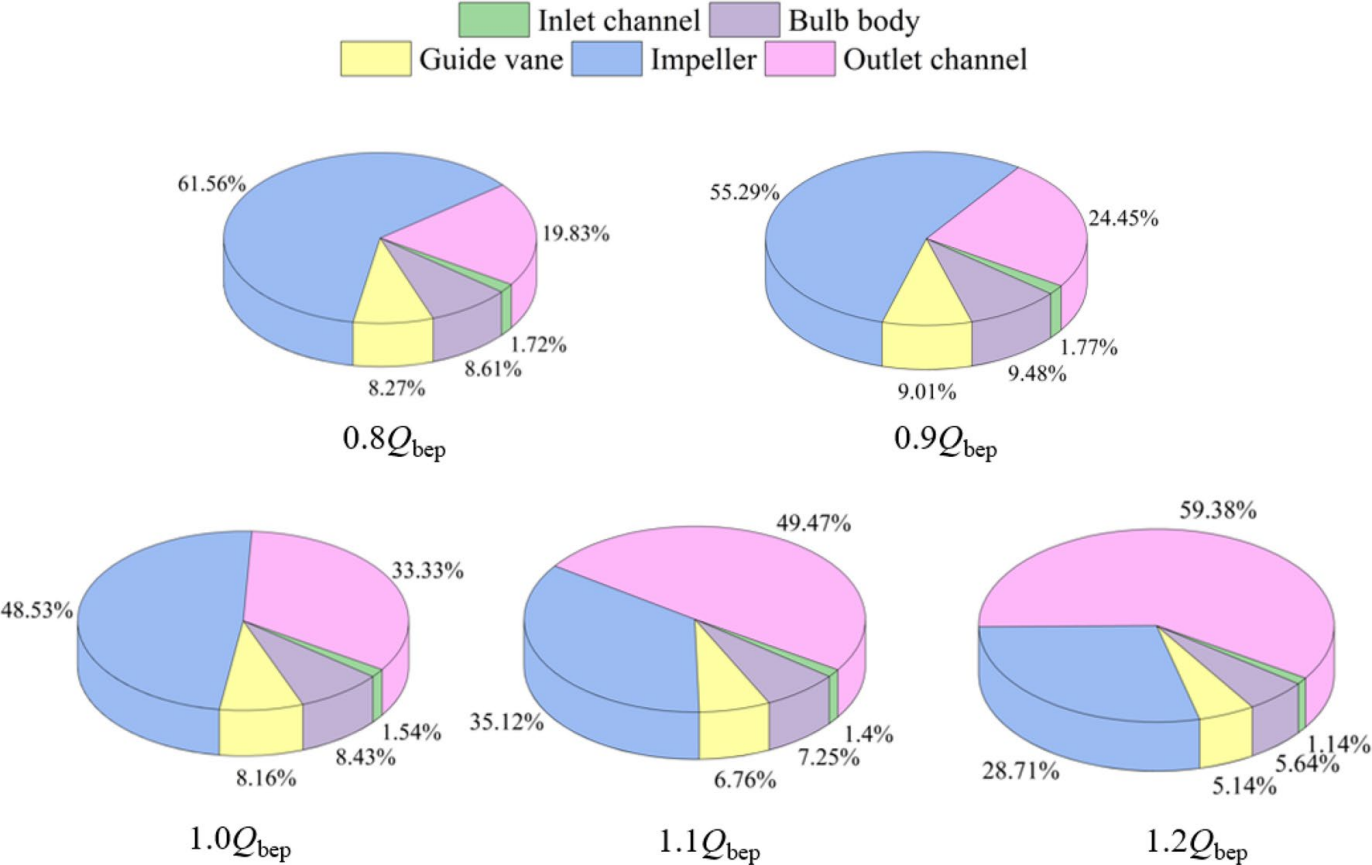
Distribution of the entropy production ratio in each region of the system under different flow conditions.

Figure 7 shows the entropy production ratio distribution of each region in the system under different flow conditions. Figure 7 shows that the entropy production ratio of the inlet is small under different flow conditions and that the sensitivity to the increase in flow rate is very weak. The entropy production ratio under the 0.8*Q*_bep_ flow condition is 1.72%, the entropy production ratio under the 1.0*Q*_bep_ flow condition is 1.54%, and the entropy production ratio under the 1.2*Q*_bep_ flow condition is 1.14%. The results show that the hydraulic loss of the inlet does not play an important role in the reverse power generation operations of the system. Therefore, an analysis of the components is omitted in the detailed analysis of the local entropy production rate in the next section. The bulb and guide vane become the water inlet components during reverse power generation operations. The sum of the entropy production ratios of the two components under different flow conditions fluctuates at approximately 10 ~ 15%. The entropy production ratios are 8.61% and 8.27% for the 0.8*Q*_bep_ flow condition, 8.43% and 8.16% for the 1.0*Q*_bep_ flow condition, and 5.64% and 5.14% for the 1.2*Q*_bep_ flow condition. When a large axial-flow pump system for reverse power generation is biased towards low flow rate conditions, the entropy production of the impeller plays an important role in the total entropy production of the system. The entropy production of the impeller is 61.56% under 0.8*Q*_bep_ flow conditions, 55.29% under 0.9*Q*_bep_ flow conditions, and 48.53% under 1.0*Q*_bep_ flow conditions, indicating that the impeller is the main source of energy loss in the system for reverse power generation under both small flow conditions and optimal conditions. When the system is biased towards high flow rate conditions, the entropy generation ratio of the impeller gradually decreases, and the entropy generation of the outlet channel begins to play an important role in the total entropy generation of the system. The entropy generation ratio of the outlet channel is 49.47% under 1.1*Q*_bep_ flow conditions and 59.38% under 1.2*Q*_bep_ flow conditions. The above results show that if the hydraulic performance of a large axial flow pump system for reverse power generation must be improved, the inlet channel, bulb body and guide vane do not need significant design updates. The energy conversion ability of the axial flow pump impeller is better under high flow rate conditions than under low flow rate conditions. The axial flow pump blade can be redesigned based on the turbine mode to improve the energy conversion ability of the impeller under the condition of a small flow rate. The entropy production of the outlet channel plays an important role in the total entropy production of the system. For large flow rates, the impact and diffusion losses of the flow in the outlet channel increase significantly. Therefore, to optimize the outlet channel, the geometric shape of the outlet channel can be redesigned, or a diversion device can be installed to significantly reduce the entropy production and total hydraulic loss of the system.

**Figure 8:**
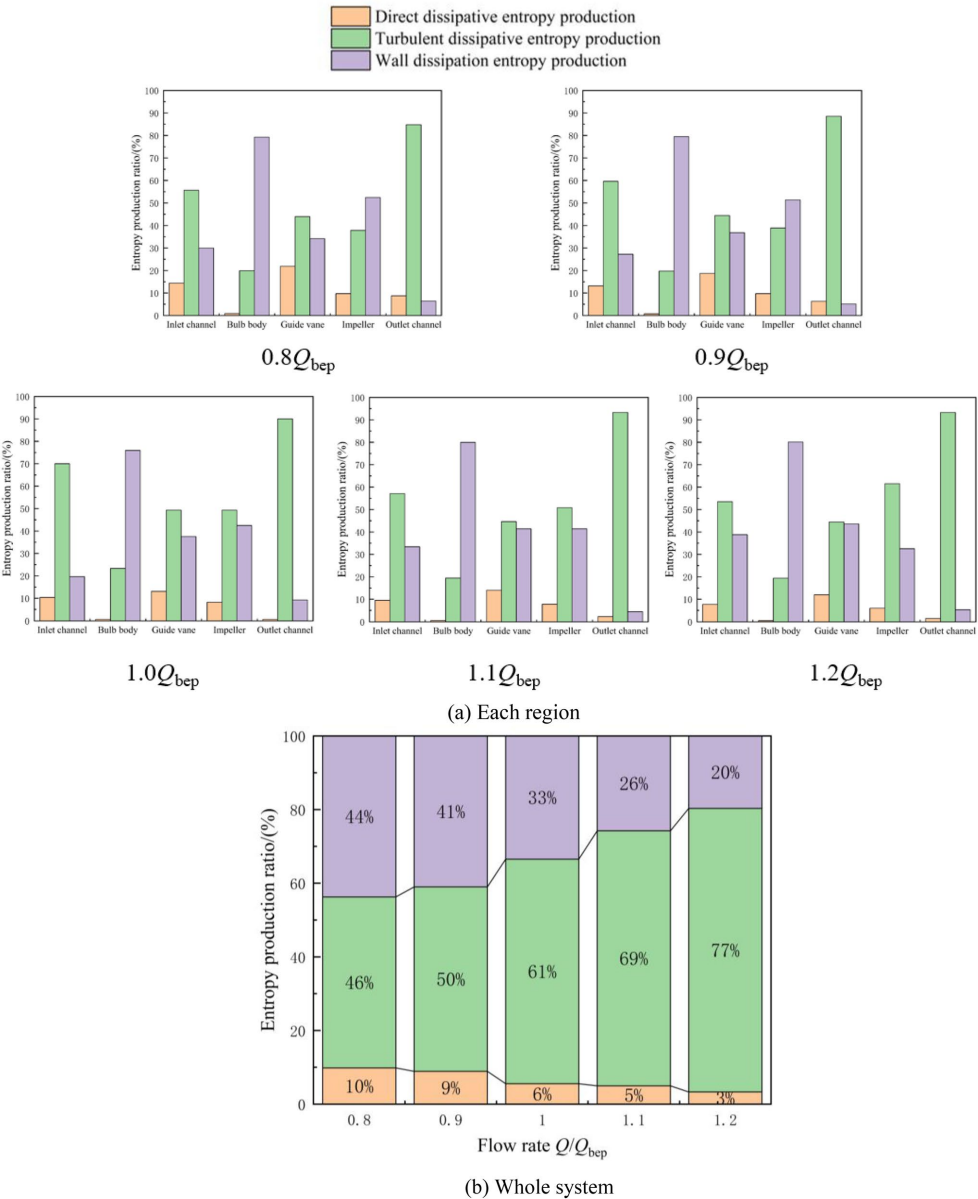
Distribution characteristics of three types of entropy generation under different flow conditions.

According to entropy production theory, the components of the total entropy production include the local entropy production and wall entropy production. The local entropy production is divided into the direct entropy production and turbulent dissipation entropy production. Among them, the turbulent dissipation entropy production is mainly related to adverse flows in the system, such as flow separation and reflux. The wall dissipation entropy production is caused by friction loss in the near-wall region. Figure 8 shows the distribution characteristics of the three types of entropy production under different flow conditions. Figure 8a shows that under different flow conditions, the proportion of the direct dissipation entropy production in the total entropy production is small in each region, and the total entropy production is dominated by turbulent dissipation entropy production and wall dissipation entropy production. The total entropy generation in the inlet passage is dominated by turbulent dissipation entropy generation under various flow conditions. The proportion of turbulent dissipation entropy generation first increases and then decreases with increasing flow rate. Under the optimal flow condition of 1.0*Q*_bep_, the proportion of the turbulent dissipation entropy production is approximately 70%, while the proportion of the wall dissipation entropy production is approximately 20%. The total entropy production of the bulb is dominated by wall entropy production under different flow conditions, and the proportion of the wall entropy production is relatively stable, while the local entropy production is relatively small. This result occurs because the bulb body is equipped with multiple large supporting blades, and the friction loss between the water flow and the large blades causes more entropy production in the wall area than in the mainstream area. Under the optimal flow condition of 1.0*Q*_bep_, the proportion of the wall dissipation entropy production is approximately 74%, while the proportion the of turbulent dissipation entropy production is approximately 22%. The turbulent dissipation entropy production and wall entropy production in the guide vane area are relatively similar under different flow conditions. Under the optimal flow condition of 1.0*Q*_bep_, the proportion of the wall dissipation entropy production is approximately 38%, while the proportion of the turbulent dissipation entropy production is approximately 49%. The total entropy generation in the impeller region is dominated by wall dissipation entropy at small flow rates. As the flow rate increases, the proportion of turbulent dissipation entropy generation gradually increases, and the total entropy generation in the impeller region begins to be dominated by turbulent dissipation entropy generation. Under the optimal flow condition of 1.0*Q*_bep_, the proportion of the wall dissipation entropy production is approximately 42%, while the proportion of the turbulent dissipation entropy production is approximately 49%. The total entropy production in the outlet channel is clearly dominated by turbulent entropy production. This result occurs because when reverse operations are carried out, the flow is discharged through the impeller without guide vane rectification, considerably impacting the diffusion intensity of the flow in the outlet channel. Under the optimal flow condition of 1.0*Q*_bep_, the proportion of the wall dissipation entropy production is approximately 8%, while the proportion of the turbulent dissipation entropy production is approximately 92%. Figure 8b shows that the proportion of the direct dissipative entropy production in the total entropy production of the system is less than 10% and that the proportion decreases with increasing flow rate. Turbulent dissipation entropy production dominates the total entropy production of the system. As the flow rate increases, the flow separation, reflux in the impeller, and diffusion of the flow in the outlet channel cause the turbulence structure of the system to increase, and the proportion of turbulent dissipation entropy production gradually increases with increasing flow rate. Under the optimal flow condition of 1.0*Q*_bep_, the proportion of turbulent dissipation entropy production in the system is 61%. The proportion of the wall dissipation entropy production in the total entropy production of the system gradually decreases with increasing flow rate, and the proportion of the wall dissipation entropy production of the system is 33% under the optimal flow condition of 1.0*Q*_bep_.

### Detailed distribution of the local entropy production rate of the bulb and guide vane

According to the analysis in the previous section, local entropy production plays a dominant role in the total entropy production of the system. As the flow rate increases, the flow separation, reflux, and diffusion of the flow in the outlet channel cause the turbulence structure of the system to increase, and the proportion of the local entropy production gradually increases with increasing the flow rate. In this chapter and sections “Detailed distribution of the impeller local entropy production rate” and “Detailed distribution of the local entropy production rate in the outlet channel”, the local entropy production rate method is used to determine the accurate location of energy loss in each region, and the energy dissipation mechanism in each region is analysed.

**Figure 9:**
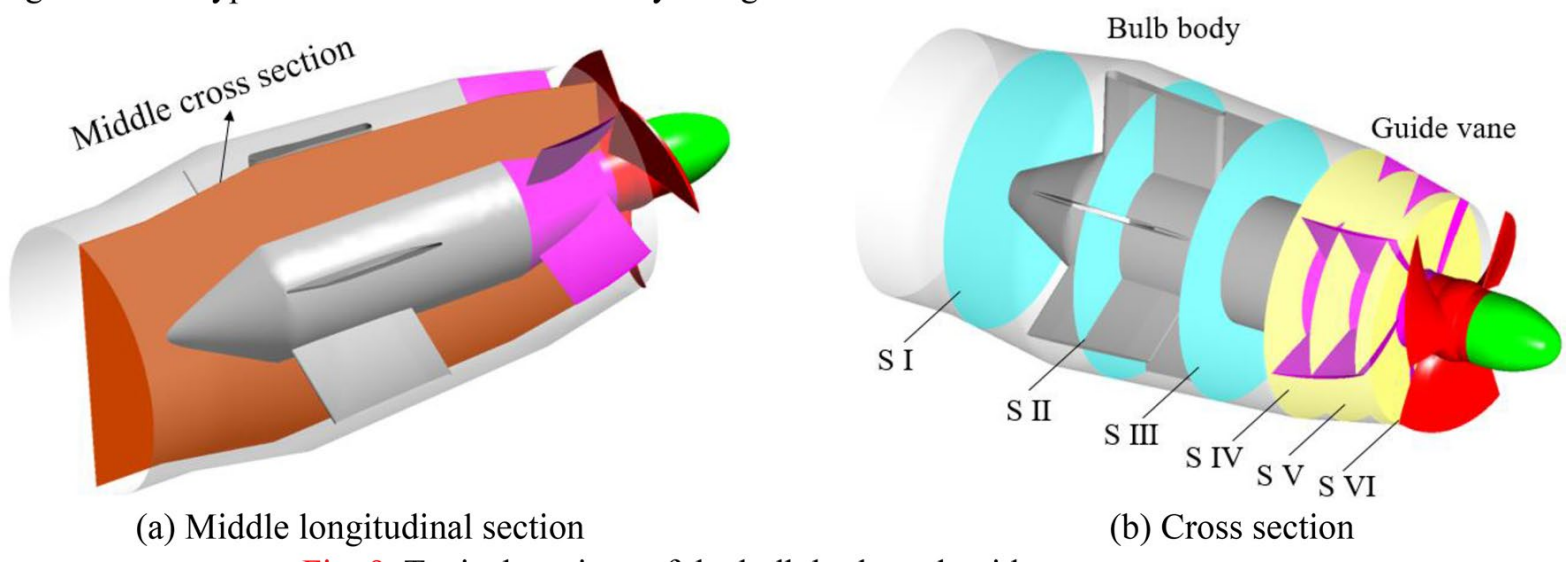
Typical sections of the bulb body and guide vane.

The bulb body and guide vane become the inlet components when the system is in reverse power generation mode, and the sum of the entropy production ratios of the two components under different flow conditions is approximately 10 ~ 15%. To study the generation mechanism of local entropy production in the bulb body and guide vane, the middle longitudinal section and six cross sections of the bulb body and guide vane were selected as typical sections to analyse the local entropy production distribution in the bulb body and guide vane along the flow direction. Figure 9 shows a schematic diagram of the typical sections in the bulb body and guide vane.

**Figure 10:**
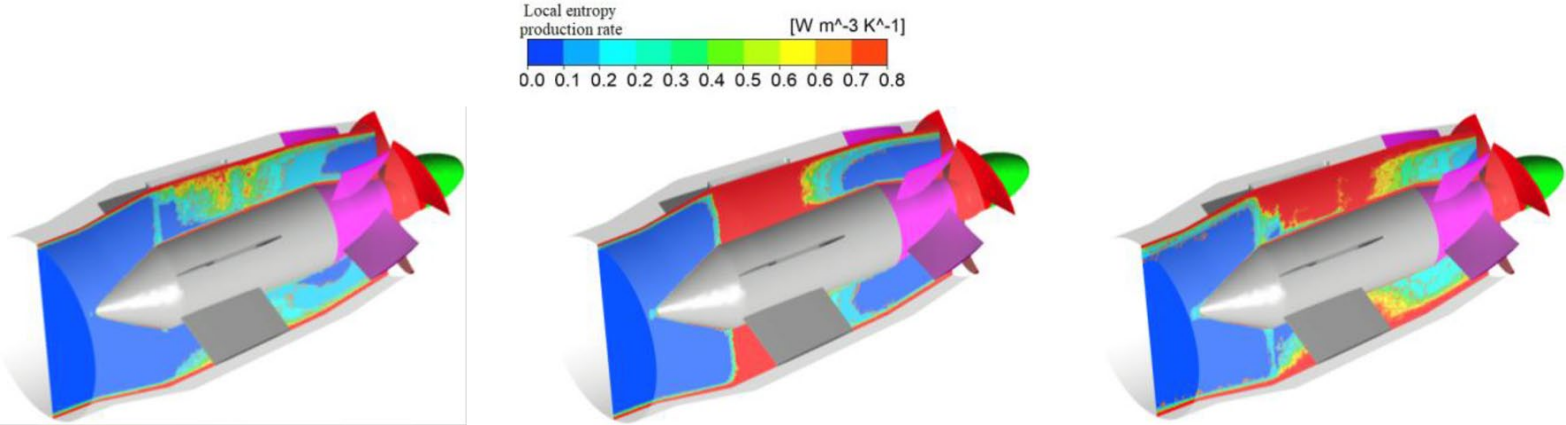
Local entropy production rate distribution in the middle longitudinal section of the bulb and guide vane.

**Figure 11:**
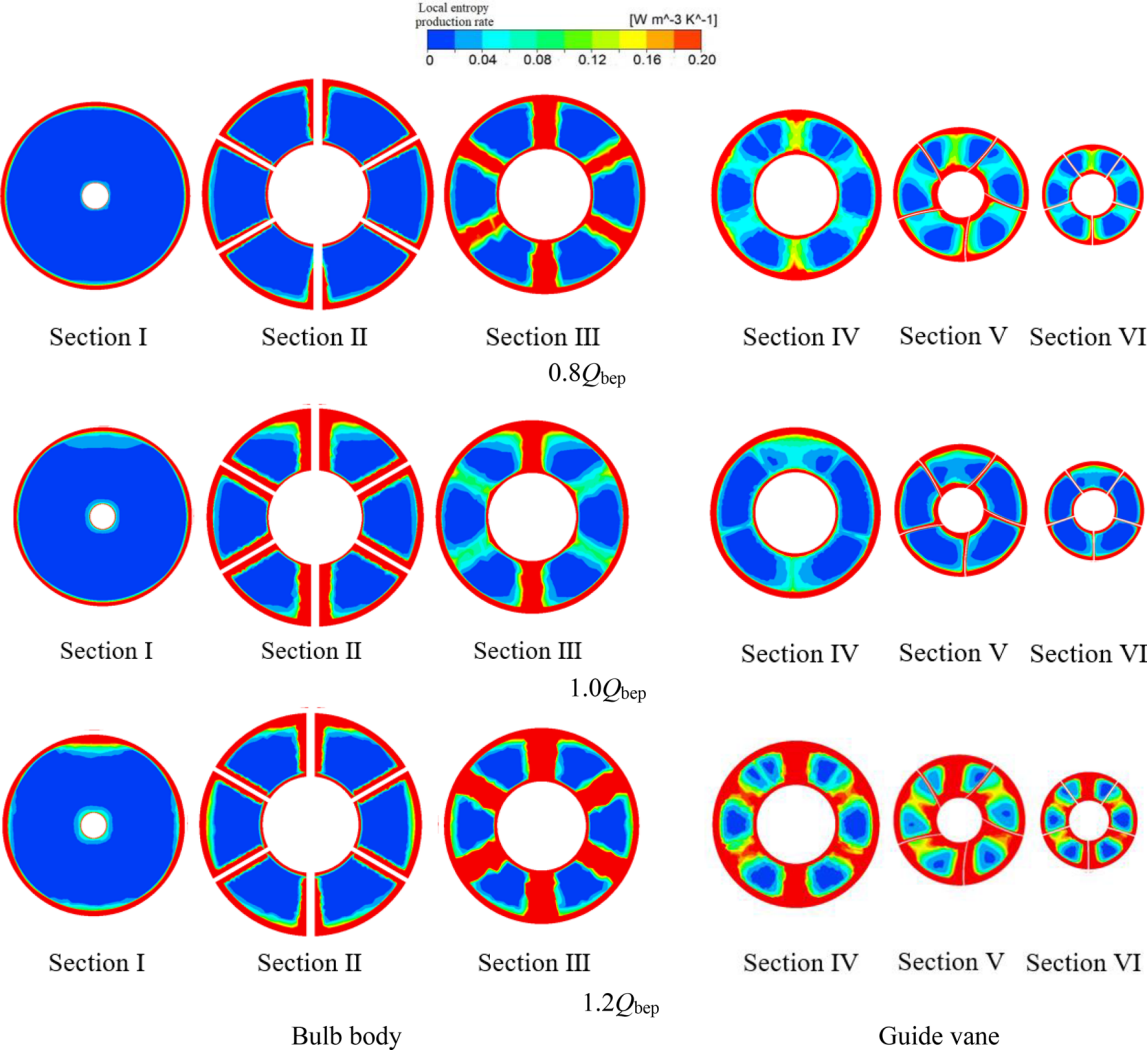
Local entropy production rate distribution of typical cross sections of the bulb and guide vane.

Figure 10 shows the distribution of the local entropy production rate in the middle longitudinal sections of the bulb and guide vane. Figure 11 shows that the water flowing into the bulb diffuses uniformly at the front end of the bulb and that the energy loss is small. Subsequently, the water collides with the head of the bulb cone, and a small region of high entropy production appears at the head of the bulb cone. When the flow moves towards the middle of the bulb, the flow is squeezed and divided by the supporting blade of the bulb, and several high entropy production areas appear in the middle of the bulb. Then, water flows out of the bulb body, and diffusion occurs in the guide vane. Similarly, as a result of the guide vane blade cutting effect, there is some entropy production in the guide vane.

Figure 11 shows the distribution of the local entropy production rate in the six cross sections of the bulb and guide vane. The following conclusions can be drawn from Fig. 11. First, six bulb-supporting blades divide the flow channel into six separate passages. Under the flow conditions of 0.8*Q*_bep_ and 1.2*Q*_bep_, the entropy production rate distribution of sections II ~ VI generally presents six fan-shaped regions with approximate distribution laws and equally spaced distributions between the high entropy production area and low entropy production area. This is because when two strands of water meet in a single channel, shear flow occurs. Due to channel compression and the shear flow, the two strands of water generate a high entropy production area at the confluence, and after the flow meets in the channel, the flow pattern is good, and the entropy production is small. Second, the entropy production rate decreases from the sidewalls of the inner and outer shells towards the centre of the water passage. This occurs because there is a significant velocity gradient near the inner shell and collision and extrusion between the sidewall and the blade, resulting in a higher entropy production rate. Near the centre of the water passage, the collision and extrusion between the edge wall and the blade are small, the flow pattern is good, and the entropy production rate is low. Third, under different flow conditions, the local entropy production rate is increased near the sidewall of the bulb and the guide vane, and with the increasing flow rate, the local entropy production rate near the sidewall clearly increases. This phenomenon can be explained by an increase in viscous stress in the boundary layer and shear stress near the wall, which has been reported in previous studies on the volute of the Francis turbine^19^.

**Figure 12:**
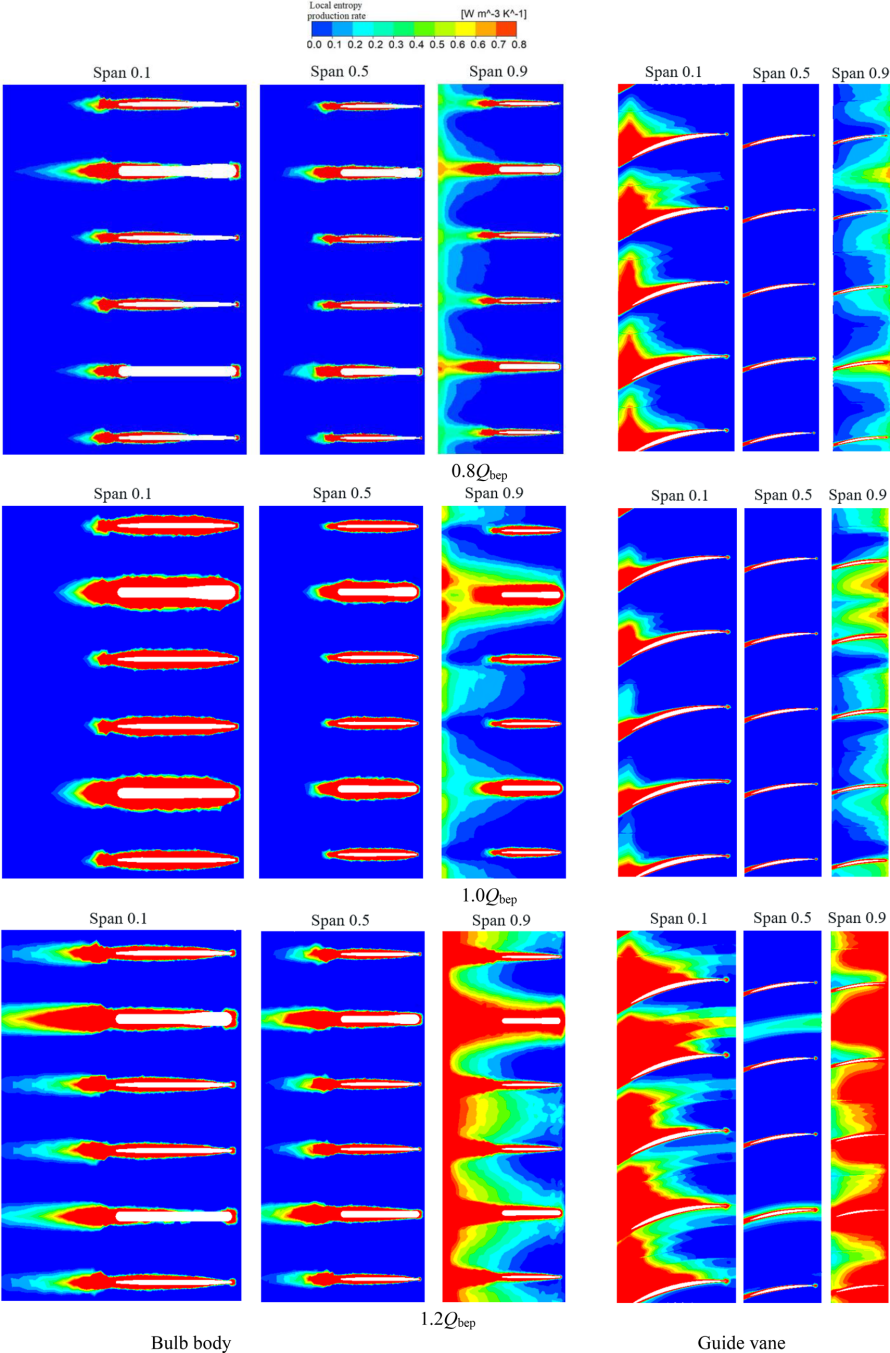
Local entropy production rate distribution of the leaf spreading section of the bulb body and guide vane.

To further analyse the generation mechanism of local entropy production in the bulb and guide vane, Fig. 12 shows the distribution of the entropy production rate in different spans of the bulb and guide vane under various flow conditions. The following conclusions can be obtained from Fig. 12. First, under different flow conditions, the range of the high entropy production area in the 0.5 span section is clearly small, especially in the guide vane. This shows that the flow pattern in the middle section of the channel is good and that the energy loss is small, which is consistent with the conclusions obtained from the analysis of Fig. 11. Second, with increasing flow rate, the impact of high-speed water flows on the head of the bulb support blade gradually increases, and banded high entropy production areas appear at the head of the bulb support blade in different span sections. Third, under the condition of a 1.0*Q*_bep_ flow rate, the water inflow angle and guide vane angle have a high degree of convergence, the shear and extrusion effects of the guide vane are weak, and the range of high entropy production in different span sections of the guide vane is clearly small. Fourth, under the condition of a 1.2*Q*_bep_ flow rate, the flow velocity in the channel increases, and the flow collides violently with the support blade and guide blade in the bulb body. The entropy production loss increases, and the range of the high entropy production area is significantly larger than that under other conditions. Moreover, under the condition of a large flow, the collision and shear flow of the water near the shell in the channel become increasingly intense, and the range of the high entropy production area in the 0.9 span section of the bulb clearly increases.

### Detailed distribution of the impeller local entropy production rate

**Figure 13:**
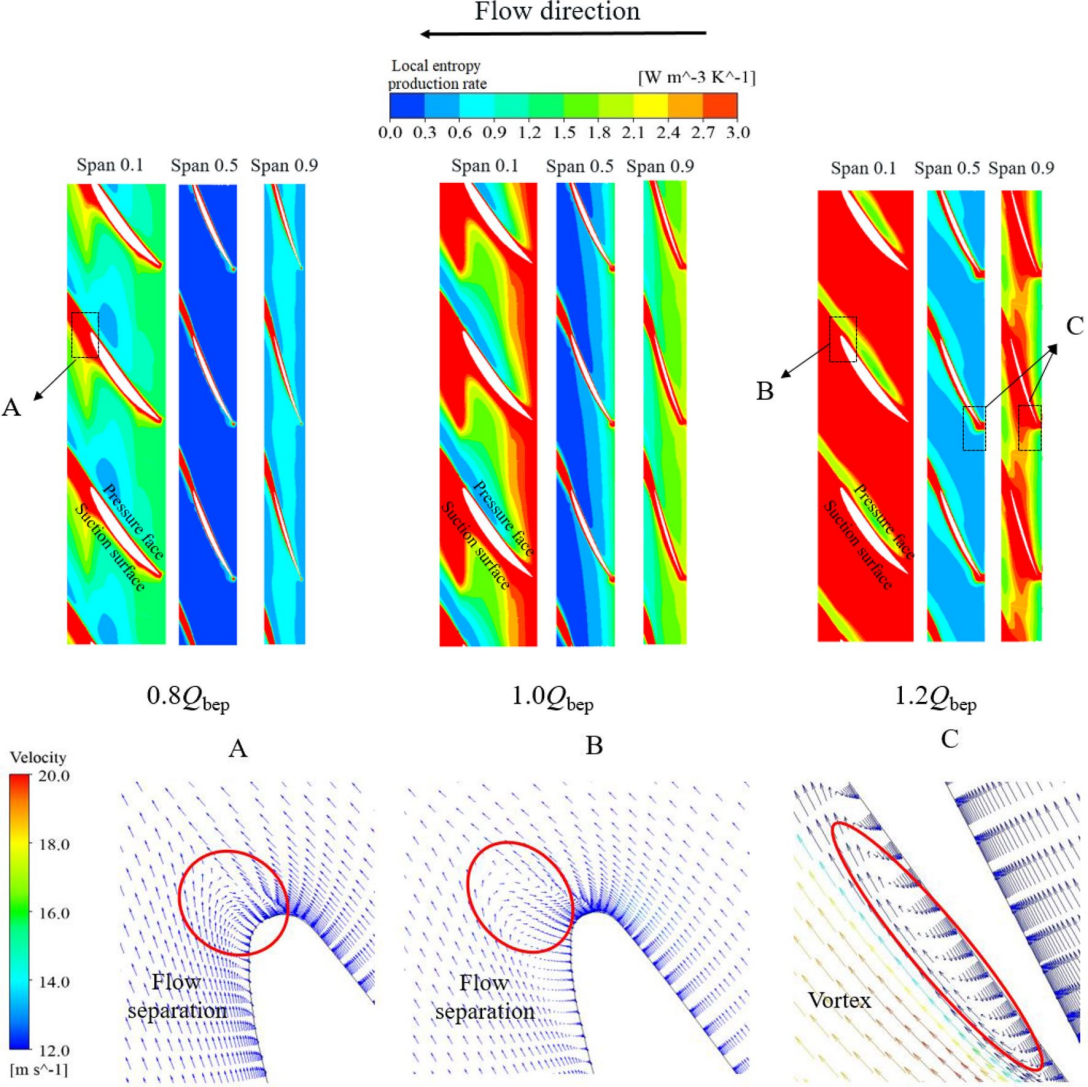
Entropy production rate distribution of different impeller spans and local velocity vector amplification diagrams.

The impeller is one of the most important components of large axial flow pump systems for reverse power generation. When a large axial flow pump system is biased towards a small flow rate, the entropy production rate of the impeller plays an important role in the total entropy production rate of the system. The entropy production rate of the impeller is 61.56% at a 0.8*Q*_bep_ flow rate, 55.29% at a 0.9*Q*_bep_ flow rate and 48.53% at a 1.0*Q*_bep_ flow rate. To study the generation mechanism of local entropy production in the impeller, different span sections of the impeller were selected to analyse changes in the local entropy production distribution in the impeller. Figure 13 shows the entropy production rate distribution and local velocity vector amplification in different spans of the impeller under various flow conditions. Figure 13 shows that under different flow conditions, the high entropy production areas of the 0.5 span section are small, while the high entropy production areas of the 0.1 span section are large. Under the flow conditions of 0.8*Q*_bep_ and 1.0*Q*_bep_, the high entropy production areas are mainly concentrated in the trailing edge of the blade suction surface and the trailing edge of the blade. This high entropy production area is closely related to flow separation and the blade wake. Under the condition of a 1.0*Q*_bep_ flow rate, the emergence of this high entropy production area is mainly related to the wake of the blade because the wake speed at the trailing edge of the blade is higher than the mainstream speed. Therefore, in the blade channel near the impeller outlet, there is a transition zone between the mainstream speed and the wake speed of the blade, resulting in a certain degree of entropy production. Under the condition of a 0.8*Q*_bep_ flow rate, flow separation becomes a dominant cause of this high entropy production area. The local velocity vector amplification diagram A shows that there is a certain range of flow separation at the trailing edge of the suction surface of the blade. This flow separation causes a low-speed zone and a corresponding separation vortex at the trailing edge of the blade. The low-speed zone and separation vortex lead to energy loss and entropy production.

Under the condition of a 1.2*Q*_bep_ flow rate, the flow rate in the pump continues to increase, and the inlet impact and outlet deviation of the blade also increase. The amplification of the local velocity vector in Fig. 13C shows that under the condition of a 1.2*Q*_bep_ flow rate, a clear vortex area forms at the front edge of the suction at the blade inlet, and this vortex area disturbs the surrounding fluid. Moreover, the local velocity vector amplification diagram B shows that at the trailing edge of the suction surface of a blade with a 0.1 span section, high-speed water flows converge at the trailing edge of the blade, causing clear flow separation. The disturbance of the vortex at the impeller inlet and the flow separation at the impeller outlet aggravate the energy dissipation of the flow under large flow conditions. The impact of the high-speed flow on adverse flows expands the range of the high entropy production area in the impeller more under the 1.2*Q*_bep_ flow condition than under the 0.8*Q*_bep_ and 1.0*Q*_bep_ flow conditions.

### Detailed distribution of the local entropy production rate in the outlet channel

**Figure 14:**
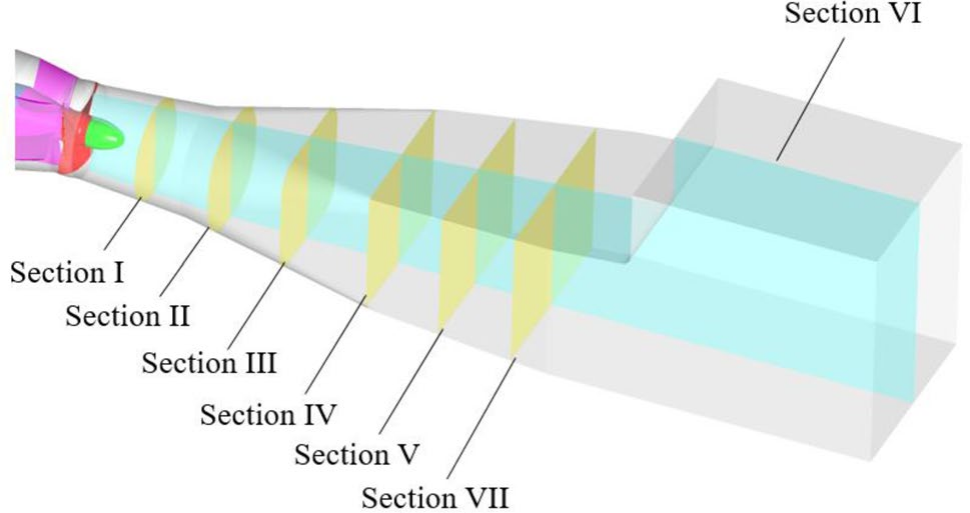
Schematic diagram of a typical cross-section selected for the outlet channel.

According to the numerical results shown in Fig. 8, the entropy production value of the outlet channel is highly sensitive to increases in the flow rate. When the system is biased towards a higher flow rate, the entropy production value of the outlet dominates the total entropy production of the system. Under a flow rate of 1.1*Q*_bep_, the total entropy production value of the outlet is 2.827 W/K, accounting for 49.47% of the total entropy production of the system. Under a flow rate of 1.2*Q*_bep_, the total entropy production value of the outlet is 5.911 W/K, accounting for 59.38% of the total entropy production of the system. To explore the energy dissipation mechanism in the outlet channel in detail, six sections of the outlet channel were selected as typical sections, as shown in Fig. 14.

**Figure 15:**
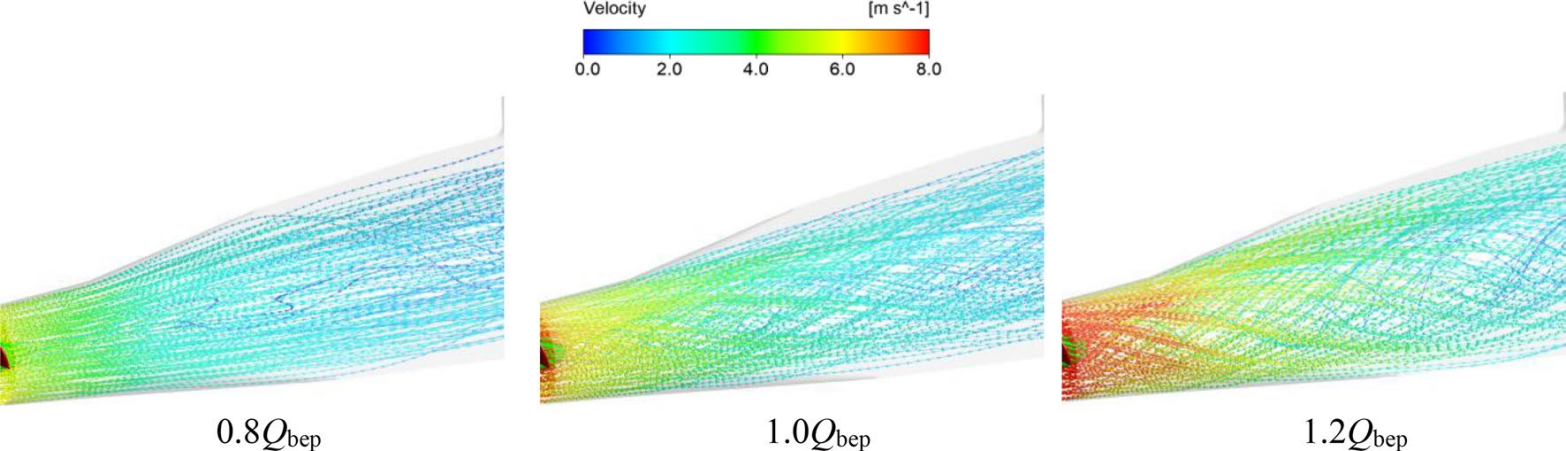
Three-dimensional velocity vector diagrams of the outlet channel under different flow conditions.

**Figure 16:**
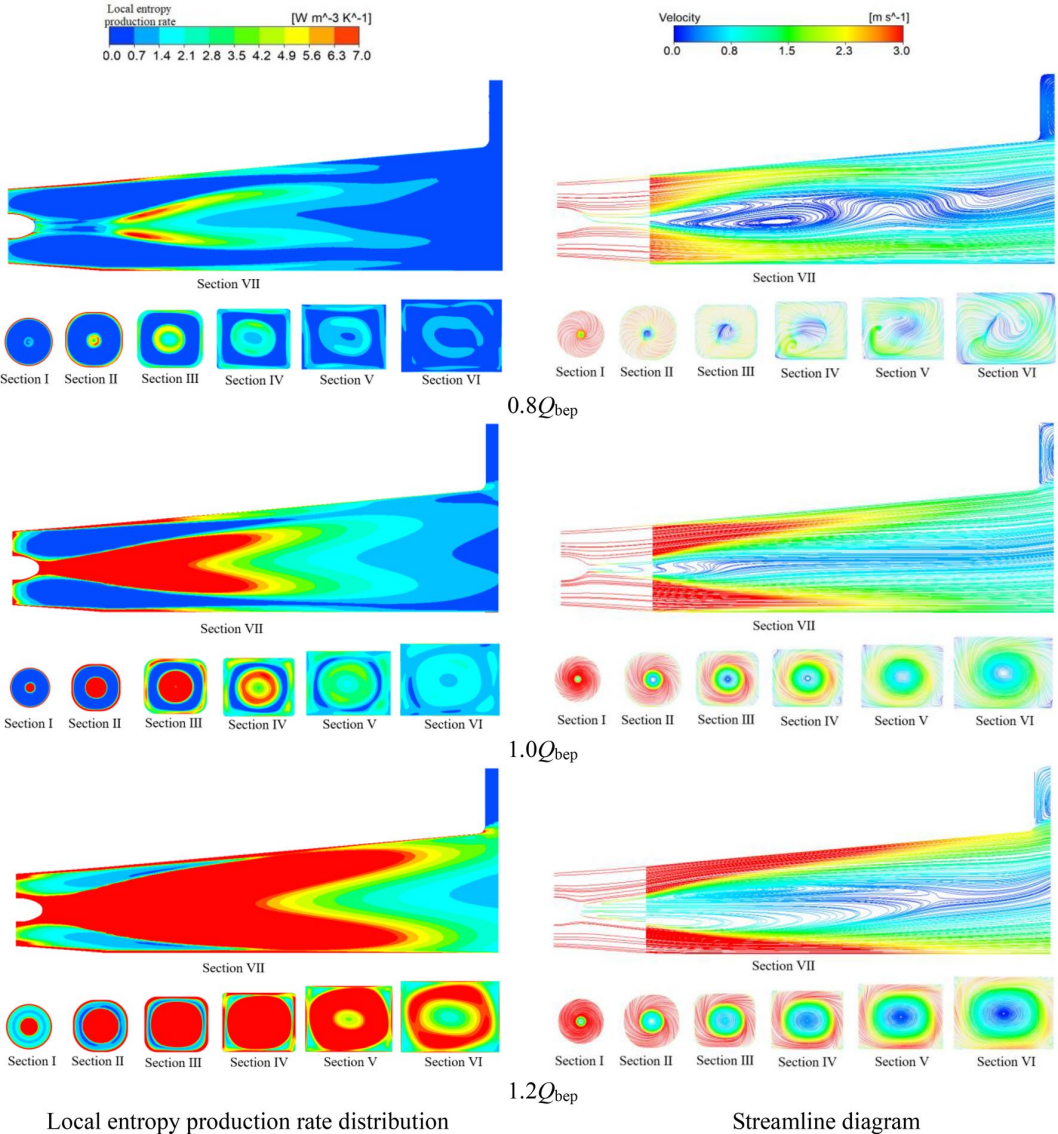
Local entropy production rate distribution and streamline diagrams of typical cross sections of the outlet channel.

Figure 15 shows three-dimensional velocity vector diagrams of the outlet under different flow conditions. Figure 16 shows the local entropy production rate distributions and streamline diagrams of typical sections of the outlet channel. The following conclusions can be obtained according to Figs. 15 and 16. First, compared with the flow conditions of 1.0*Q*_bep_ and 1.2*Q*_bep_, the local entropy production rate in the outlet channel is smaller under the flow condition of 0.8*Q*_bep_, and the range of the high entropy production area of each typical section is also smaller. According to the three-dimensional velocity vector diagrams and the streamline diagrams of typical sections, there are some adverse flows, such as vortices and cross flows, in the outlet channel; however, there are no obvious swirls at the outlet of the impeller, and the rotation strength of the flow in the channel is low. Thus, adverse flow, such as vortices and cross flows, is the main source of entropy production in the outlet channel under small flow conditions. Second, it is worth noting that there are no clear vortices, cross flows or other adverse flows in the outlet channel under the high efficiency flow condition of 1.0*Q*_bep_; however, compared with the small flow condition, the entropy production in the outlet channel is increased. The main reason for this phenomenon is that with increasing flow velocity, the impeller outlet forms a certain degree of swirl, and the enhancement of the flow rotation in the outlet channel and the increase in the turbulence structure cause the high entropy production area in the outlet channel to expand under high efficiency point conditions. Third, when the flow velocity in the system exceeds the efficient point velocity, the entropy generation rate in the outlet channel increases sharply due to the eddy current near the middle of the outlet channel. Under the large flow condition of 1.2*Q*_bep_, the range of the high entropy production area of a typical section of the outlet channel increases significantly, and the high entropy production area is mainly distributed near the middle of the outlet channel, namely, near sections II, III, IV and V. According to the three-dimensional velocity vector diagrams and the streamline diagrams of the typical sections, the swirling velocity at the outlet of the impeller increases significantly under large flow conditions, and the high-speed swirling flow caused by the rotation of the impeller spirals forward in the outlet channel. The high-frequency eddy current generated by the rotation of the PAT runner impacts and dissipates in the vicinity of the middle of the outflow channel, resulting in a significant increase in the turbulence structure in the middle of the outflow channel, which leads directly to a surge in the total entropy generation rate of the outflow channel under large flow conditions. This result is consistent with the flow analysis in the draft tube of a very low head axial flow turbine in Ref.^27^.

## Conclusions

To reveal the energy loss mechanism of a large axial flow pump system in reverse power generation operations and to provide theoretical guidance for the application and promotion of such renewable energy projects, in this paper, the entropy generation method is used to investigate the accurate size and distribution of the mechanical energy dissipation of the components of a large axial flow pump system (inlet channel, bulb body, guide vane, impeller and outlet channel) for reverse power generation, and the changes and causes of the energy dissipation distribution of each component are analysed in depth under different flow conditions. The main conclusions are as follows:

1. The proportion of direct dissipative entropy generation in the total entropy generation of the system is less than 10%, and this proportion decreases with increasing flow rate. The turbulent dissipation entropy generation always dominates the total entropy generation of the system, and with increasing flow rate, the flow separation, backflow in the impeller, and diffusion of the water flow in the outflow channel lead to an increase in the turbulence structure of the system, as well as an increase in the proportion of turbulent dissipation entropy generation. Under the optimal flow rate of 1.0*Q*_bep_, the proportion of turbulent dissipation entropy production in the system is 61%, and the proportion of dissipative entropy production on the system wall is 33%.
2. The bulb and the guide vane become the inlet components when the system is in reverse power generation mode. Under different flow conditions, the sum of the entropy production ratio of the two components is approximately 10–15%. The entropy production rates of the bulb and guide vane gradually decrease from the sidewall of the inner shell and outer shell to the centre of the water passage. The local entropy generation rate is higher at the sidewall of the bulb and guide vane, and with increasing flow rate, the local entropy generation rate at the sidewall clearly increases.
3. When the large axial flow pump system operates at a small flow rate, the entropy production rate of the impeller dominates the total entropy production rate of the system. The entropy production rate of the impeller is 61.56% at a 0.8*Q*_bep_ flow rate, 55.29% at a 0.9*Q*_bep_ flow rate, and 48.53% at a 1.0*Q*_bep_ flow rate. Under the conditions of a low flow rate and a high efficiency point flow rate, the high entropy generation area is mainly concentrated at the trailing edge of the blade suction surface and the trailing edge of the blade, and it is mainly related to the flow separation of the trailing edge of the blade suction surface and the blade wake. Under the condition of a high flow rate, the energy dissipation of the water flow intensifies due to the disturbance of the vortex at the inlet and the flow separation at the outlet of the impeller, and the range of the high entropy production area in the impeller increases.
4. The entropy production value of the outlet channel is more sensitive to increases in the flow rate than other components of the system. When the system is biased towards a higher flow rate, the entropy production value of the outlet channel dominates the total entropy production of the system, accounting for 49.47% of the total entropy production of the system under the condition of a 1.1*Q*_bep_ flow rate and 59.38% of the total entropy production of the system under the condition of a 1.2*Q*_bep_ flow rate. Under the condition of a low flow rate, the local entropy production rate in the outlet channel is low, and the range of the high entropy production area in each typical section is small. Adverse flows, such as vortices and cross flows, are the main source of entropy production in the outlet channel under the condition of a low flow rate. When the flow velocity in the system exceeds the flow velocity at the highest efficiency point, the entropy generation rate in the outflow channel increases sharply due to the high-speed rotating vortex near the middle of the outlet channel.
5. When a large axial flow pump station system performs reverse power generation operations, the inlet channel, bulb body and guide vane do not need to be significantly redesigned. The energy conversion ability of the axial flow pump impeller is poor under small flow conditions. The axial flow pump blade can be modified based on turbine mode to improve the energy conversion ability of the impeller under small flow conditions. The entropy production of the outlet channel plays an important role in the total entropy production of the system. Therefore, redesigning the geometric shape of the outlet channel or installing a diversion device in the outlet channel to optimize the outlet channel can significantly reduce the entropy production and total hydraulic loss of the system.

## Data Availability

The datasets used and/or analysed during the current study available from the corresponding author on reasonable request.

## List of symbols

*C*_*p*_: Pressure pulsation coefficient
*f*: Frequency (s^−^^1^)
F: Frequency after the Fourier transform (s^−^^1^)
*g*: Local acceleration of gravity (m/s^2^)
*H*_exp_: Experimental head (m)
*k*: Turbulent kinetic energy
*M*: Impeller torque (N m)
*N*_*F*_: Multiple of the rotational frequency
*P*: Transient pressure (Pa)
*Q*: Flow rate of the model pump system (m^3^/s)
*Q*_*d*_: Design flow rate of the pump under pumping conditions
*Q*_bep_: Flow corresponding to the highest efficiency point under reverse power generation conditions
*S*: The invariant of the strain rate
*t*: Time (s)
T: Temperature (K)
*β*_*1*_: Blade placement angle
*ρ*: The density of the flow (kg/m^3^)
*ω*: Turbulence frequency
*η*: Efficiency (%)
*η*_exp_: Experimental efficiency (%)
$\rho_{m}$: The mixed density (kg/m^3^)
$u_{j}$: Velocity component in the j direction (m/s)
$\mu_{t}$: The turbulent viscosity (Pa s)
$$F_{1}$$,$$F_{2}$$: Mixed functions
$D_{\omega }$: Dissipation term in the ${\upomega }$-equation
$\overrightarrow {\tau }$: Wall shear stress (Pa)
$u_{j}$: Velocity near the wall (m/s)
$\mathop {S_{{\overline{D} }}^{{\prime \prime \prime}} }\limits^{ \cdot }$: Velocity average entropy production rate (W m^−3^ K^−3^)
$\mathop {S_{{D^{'} }}^{{\prime \prime \prime }} }\limits^{ \cdot }$: The entropy production rate of the velocity fluctuations (W m^−^^3^ K^−^^3^)
$\mathop {S_{W}^{{\prime \prime \prime}} }\limits^{ \cdot }$: Entropy production rate near the wall (W m^−3^ K^−3^)

## Abbreviations

CFD: Computational fluid dynamics
SST: Shear-stress transport
